# Assessing low-frequency oscillations in cerebrovascular diseases and related conditions with near-infrared spectroscopy: a plausible method for evaluating cerebral autoregulation?

**DOI:** 10.1117/1.NPh.5.3.030901

**Published:** 2018-09-18

**Authors:** Adam Vittrup Andersen, Sofie Amalie Simonsen, Henrik Winther Schytz, Helle Klingenberg Iversen

**Affiliations:** a Rigshospitalet, Department of Neurology, Glostrup, Denmark; b University of Copenhagen, Department of Clinical Medicine, Copenhagen, Denmark

**Keywords:** cerebral autoregulation, near-infrared spectroscopy, low-frequency oscillations, cerebrovascular diseases, risk of stroke

## Abstract

**Background:** Cerebral autoregulation (CA) is the brain’s ability to always maintain an adequate and relatively constant blood supply, which is often impaired in cerebrovascular diseases. Near-infrared spectroscopy (NIRS) examines oxygenated hemoglobin (OxyHb) in the cerebral cortex. Low- and very low-frequency oscillations (LFOs≈0.1  Hz and VLFOs≈0.05 to 0.01 Hz) in OxyHb have been proposed to reflect CA.

**Aim:** To systematically review published results on OxyHb LFOs and VLFOs in cerebrovascular diseases and related conditions measured with NIRS.

**Approach:** A systematic search was performed in the MEDLINE database, which generated 36 studies relevant for inclusion.

**Results:** Healthy people have relatively stable LFOs. LFO amplitude seems to reflect myogenic CA being decreased by vasomotor paralysis in stroke, by smooth muscle damage or as compensatory action in other conditions but can also be influenced by the sympathetic tone. VLFO amplitude is believed to reflect neurogenic and metabolic CA and is lower in stroke, atherosclerosis, and with aging. Both LFO and VLFO synchronizations appear disturbed in stroke, while the former is also altered in internal carotid stenosis and hypertension.

**Conclusion:** We conclude that amplitudes of LFOs and VLFOs are relatively robust measures for evaluating mechanisms of CA and synchronization analyses can show temporal disruption of CA. Further research and more coherent methodologies are needed.

## 1 Introduction

Cerebral autoregulation (CA) of cerebral blood flow (CBF) is the process in which the cerebral vasculature maintains a relatively constant blood flow despite changes in perfusion pressure.^1^ CA is impaired in several neurological diseases.^2^ The mechanisms and methods of investigating the CA have been a subject of research and discussion ever since CA as a concept was conceived.^3^ Both large arteries and small arterioles contribute significantly to vascular resistance in the brain and studies have shown that the large extracranial vessels (internal carotid and vertebral) and intracranial pial vessels contribute to around 50% of cerebral vascular resistance (CVR).^4^ In recent years, some of the most frequently used methods in studying CA in humans have been transcranial Doppler (TCD) and near-infrared spectroscopy (NIRS). TCD assesses blood velocity in major cerebral vessels, whereas NIRS detects cerebral cortical hemoglobin oxygenation and thereby changes in microcirculatory blood volume and flow. Both techniques can examine low-frequent hemodynamic parameters with high temporal resolution, but NIRS offers distinct advantages in being easy to apply, operator-independent and directly measuring hemodynamics in the cortical region of interest.

The aim here was to systematically review studies on low- frequency oscillations (LFOs) and very low-frequency oscillations (VLFOs) measured by NIRS in cerebrovascular diseases and related conditions to assess CA.

## 2 Background

### 2.1 Cerebral Autoregulation

**Fig. 1:**
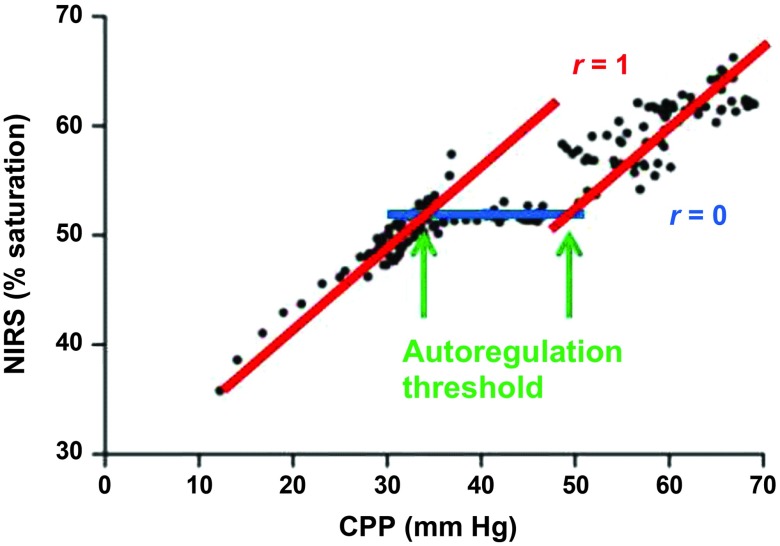
Near-infrared spectroscopy (NIRS)-derived oxygen saturation versus cerebral perfusion pressure (CPP), depicting the cerebral autoregulation curve. With permission from Moerman et al.^5^ under Creative Commons Attribution License. Original data from Brady et al.^6^

The CA ensures that CBF is maintained at a relatively constant level within large variations of arterial blood pressure (ABP) and thus the cerebral perfusion pressure (CPP)^1^ (Fig. 1):

$$\mathrm{CBF}=\frac{\mathrm{CPP}}{\mathrm{CVR}}=\frac{\mathrm{ABP}-\text{intracranial pressure}}{\mathrm{CVR}}.$$ (1)

This serves as a protective measure against ischemia and failure could possibly result in pallor, sweating, confusion, dizziness, or syncope.^7^^,^^8^ In acute brain injury, where the tissue is particularly sensitive, hypoperfusion can lead to progression of the damages due to impaired CA^7^^,^^8^ and several neurologic disorders do exhibit impaired or changed CA.^2^ In the opposite end of the arterial blood pressure (ABP) spectrum, CA also protects against chronic hypertension and hypertensive encephalopathy.^9^^,^^10^

Several factors influence CA. The myogenic mechanism is a direct effect in smooth muscle due to changes in transmural pressure mostly active in the small vessels of the brain.^1^^,^^11^^–^^13^ The endothelium has a prominent role by the release of dilatory and constrictory substances in addition to the direct mechanical response.^1^^,^^13^^,^^14^ Though no one single chemical agent has yet been identified, a metabolic response is observed with neuronal activation in local or global areas.^1^^,^^11^^,^^13^^,^^15^^,^^16^ Autonomic nerve fibers richly innervate the cerebral vessels, especially the larger arteries and activation of the sympathetic nervous system (SNS) pushes the limits of the autoregulation plateau up higher and vice versa.^1^^,^^11^^,^^13^^,^^17^^,^^18^ The level of the partial pressure of carbon dioxide in arterial blood (${\mathrm{PaCO}}_{2}$) also modifies CA considerably.^1^^,^^19^ Hypercapnia increases CBF by vasodilation, which also narrows the autoregulatory plateau with changes of both the upper and lower CA limit. Hypocapnia decreases CBF by vasoconstriction, but does not affect the lower limit of CA, while uncertainty remains concerning the upper limit. Furthermore, other physiological systems such as the renin–angiotensin–aldosterone system and pharmacologic substances can modify the complex nature of CA. The distribution of resistance within the microvasculature is a contentious area of research and especially the role of pericytes and capillary resistance.^20^^,^^21^

The research on CA has developed in two main fields. Static CA is the determination of the autoregulatory limits of ABP under different relatively steady circumstances. Dynamic CA includes investigations in both spontaneous physiological fluctuations in ABP and CBF, but also the CBF response to sudden changes in ABP, e.g., when changing body position or following deflation of a cuff in order to quickly pool or release blood from one or more extremities. The analysis of low-LFOs is a part of dynamic CA, which we will be reviewing in the following.

### 2.2 Mayer Waves and Low-Frequency Oscillations

**Fig. 2:**
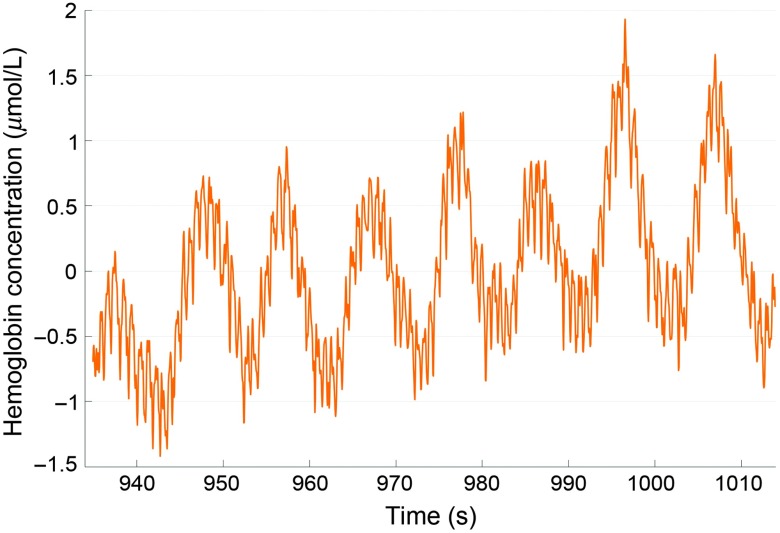
OxyHb oscillations at cardiac frequency (1 Hz) and at M-wave frequency (0.1 Hz) enhanced with deep breathing at 0.1 Hz. Unpublished data from healthy elderly woman.

Mayer waves (M-waves) is a phenomenon of slow spontaneous ABP oscillations observed in 1876 by Mayer in rabbits.^22^ Slower than ABP oscillations of cardiac (≈1  Hz) and respiratory rhythms (≈0.2 to 0.5 Hz), these M-waves has different frequencies across species, but about 0.1 Hz when observed in humans (Fig. 2). M-waves are most often termed LFOs. The oscillations in this frequency are believed to reflect sympathetic nervous activity (SNA) as they are enhanced with activation of the SNS and SNA have, therefore, often been attached to the definition of LFOs.^23^ Their origin is thought to be the vasomotor tone of blood vessels synchronous throughout the body,^23^^,^^24^ but the driving mechanism behind the rhythmicity of the oscillations has not yet been fully established.

#### 2.2.1 Pacemaker theory and baroreceptor reflex theory

The pacemaker theory, primarily based on animal studies, suggests that the central nervous system contains a pacemaker that is responsible for the rhythmicity of LFOs.^23^ Studies have shown slow SNA and ABP rhythms in the LFO frequency despite the lack of sensory inputs from peripheral structures in animals that have undergone surgical and/or medical denervation.^25^^–^^28^ In tetraplegic humans with traumatic spinal cord lesions, the results have been conflicting.^29^^–^^31^ While one study showed consistent LFOs,^31^ other studies failed to detect LFOs in some^29^ or all subjects^30^ perhaps due to different levels of the spinal lesions or differences in tetraplegia durations as one study proved significantly increased LFOs 6 months after the initial examination.^29^

The baroreflex theory originates from the work of Guyton and Harris.^32^ The contribution of the baroreceptor reflex to the genesis of LFOs has since been confirmed in numerous studies of animals undergone surgical sinoaortic baroreceptor denervation^33^^–^^35^ showing either strong attenuation or abolishment of LFOs. Deactivation of the baroreceptor reflex with alpha-adreno blockers shows a similar trend in animals^36^^–^^38^ and humans.^39^^,^^40^ These studies support that the LFOs are caused by vasomotor tone, because alpha-adrenergic antagonists block the sympathetic effect on vasomotion.^41^

#### 2.2.2 Amplitude

There are only a limited amount of studies focusing on the amplitude of the LFOs. In animal studies the amplitude follows the mean level of SNA when subjected to stimulations altering the sympathetic tone.^42^^–^^45^ Human studies show the same tendency^46^^–^^50^ though only in individuals and not across groups with different SNA levels^51^ perhaps due to age differences in the vasculature and regulation thereof. The reproducibility of LFO amplitudes is high over short-term, but low over long-term,^52^ though this could be influenced by several factors including overall stress level.^49^ It has been suggested that the LFO amplitudes indicates reflectory local myogenic activity^53^ in the terminal arteriole smooth muscle cells,^54^ and is influenced by sympathetic control mechanisms.^55^ It is thought that the local myogenic response deals with small changes in systemic pressure changes, while the SNS responds to larger changes,^56^ but the exact relationship remains uncertain.

### 2.3 Very Low-Frequency Oscillations

Oscillations at an even lower frequency have been observed in a range distinctly below LFOs (0.05 to 0.01 Hz). Originally observed in intracranial pressure by Lundberg,^57^ these oscillations were also seen in ABP and velocity of the medial cerebral artery ($V_{\mathrm{MCA}}$)^58^^,^^59^ and termed VLFOs. The oscillations in these different parameters have been shown to be connected and several autoregulatory mechanisms have been proposed to explain this connection.^60^^–^^64^

The origin of VLFOs is thought to be generated from a central pacemaker because of the relation between different oscillating parameters mentioned above, but also because of the interhemispheric synchronicity.^59^^,^^65^^–^^68^ Rhythmic changes in breathing and thereby changes in ${\mathrm{PaCO}}_{2}$ have also been shown to attribute to VLFOs as they occur at about the same frequency (≈0.03  Hz) and correlates well with blood-oxygenation-level-dependent (BOLD) signals for CBF both under steady state^69^^,^^70^ and with neural activity.^71^ CPP values are described to have influence on the amplitude and frequency of VLFOs,^72^ while they remain relatively independent of changes in ABP. Intaglietta et al.^54^ proposed that they stem from large arterioles under neurogenic innervation^73^ and thus sympathetic control.^74^ In some cases, the VLFOs are divided into two frequency ranges and Stefanovska et al.^53^ showed that while the upper VLFO range is endothelial independent and probably relies on the neurogenic activity in large arteries, the lower VLFO range is endothelial-related, and therefore, connected to metabolic changes in the microvessels (Table 1).

**Table 1:** Physiological ABP oscillations.

Oscillation	Frequency (Hz)	Factors considered to determine amplitude
Cardiac	≈1	Heart pumping
Respiratory	≈0.25	Respiration changing intrathoracic pressure
LFOs	≈0.1	Local myogenic activity in terminal arteriole
		Influenced by sympathetic tone and control mechanisms
Upper VLFOs	≈0.05	Neurogenic SNS activity in large arteries
		Spontaneous modulation of respiration frequency changing ${\mathrm{PaCO}}_{2}$ level
		Independent of endothelium
Bottom VLFOs	≈0.01	Metabolic activity in microvessels
		Dependent on endothelium

### 2.4 Physiological Function of Oscillations

The physiological purpose of the oscillations low in the frequency spectrum remains intangible. Theories of cyclic nitric oxide (NO) release from the endothelium beneficial to organ function^75^ and a possible nutritive function^76^ from animal studies have not been confirmed. The possibility remains that the oscillations are an observed epiphenomenon with no functional purpose.^77^

### 2.5 LFOs and VLFOs in Evaluating Cerebral Autoregulation

Similarities in existing knowledge of CA and origins of LFOs and VLFOs indicate an association. Giller originally proposed the method of coherence analysis between systemic and cerebral perfusion oscillations in the low-frequency spectrum to evaluate dynamic CA as the oscillations would be altered with intact CA and relatively unaltered with impaired CA.^60^ Diehl et al.^78^ contrived the model of dynamic CA being a biologic control system working as a high-pass filter transmitting high-frequent ABP-oscillation unfiltered to CBF, while LFOs are filtered and only passed through to CBF partially. Considering this, measures of gain and phase shift were suggested to quantify this transmission and thus CA.

Katura et al.^79^ demonstrated that while an substantial amount of the cerebral vessel LFOs can be attributed to systemic LFOs, more than half cannot, indicating that the origin of cerebral LFO may lie in the autoregulation of CBF rather than systemic cardiovascular regulation.

While the high-pass filter model is not perfect, it does imply two important features of LFOs that correlate with CA: the amplitude and the synchronization. Especially, the latter has been analyzed in different ways as healthy people exhibit highly synchronized oscillations as an expression of well-functioning central origin mechanisms and thereby intact CA. Feasibly, the amplitude on the other hand could in part be ascribed to the magnitude of autoregulatory mechanisms.

### 2.6 Investigating Cerebral Low-Frequency Oscillations

Spontaneous LFOs in cerebral vessels can be examined through methods such as ultrasonic TCD ^60^ and NIRS,^80^ but they can also be accentuated by stimulations of the same frequency.^78^^,^^80^ Both methods are equipped to examine the cerebral blood circulation over time with high temporal resolution and can be applied under various conditions and stimulations that cannot be examined by more confined imaging modalities such as BOLD-magnetic resonance imaging (MRI). While TCD estimates the velocity in larger cerebral arteries such as the MCA, NIRS investigates the cerebral tissue including all vascular compartments though quantitatively reflecting the microvasculature.^81^ The TCD method has dominated the CA research over NIRS, despite TCD being challenged with certain assumptions and limitations. To assess CBF from measurements of $V_{\mathrm{MCA}}$, it is assumed that the large arteries have a constant diameter while studies have shown that the opposite is the case.^82^^,^^83^ Additionally, 5% to 20% of the population cannot be assessed properly due to a poor acoustic window.^84^ Although NIRS has other limitations and assumptions, at least the method offers a direct measure of local CBF.

**Fig. 3:**
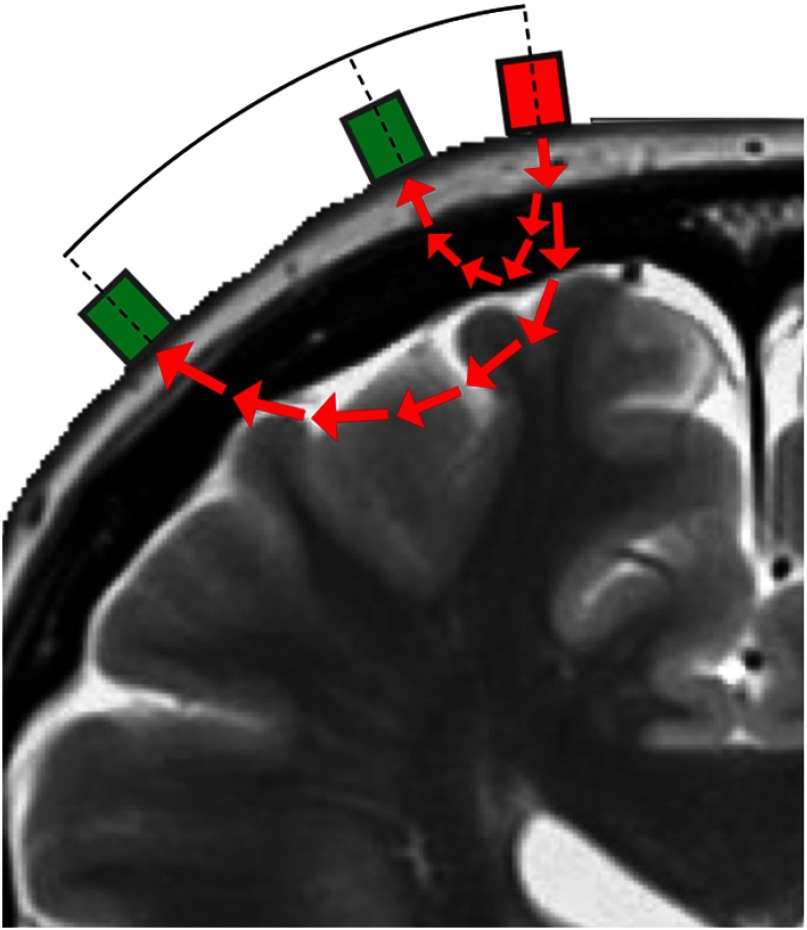
NIRS with short separation.

The method is simple to understand in general.^81^ Infrared light of certain wavelengths between 650 and 950 nm is passed from the skin through the extracranial layers and the cortex of the brain. The light passes through human tissue being absorbed by water, fat, melanin, and both oxygenated (OxyHb) and deoxygenated hemoglobin (DeoxyHb). On the skin, adjacent to the light source, detectors are that pick up the infrared signal emanating from the body. Since the levels of water, fat, and melanin remain constant, the signal can then be calculated into dynamic OxyHb and DeoxyHb concentrations by a modified version of the Beer–Lamberts law. To filter the extracranial signals from the intracranial signals, researchers often use multiple light detectors with different distances from the light source and separate the signals from each other to get the purest intracranial signal possible^85^ (Fig. 3).

In the current study, we will address the NIRS modality called continuous wave, which is not appropriate to calculate an exact hemoglobin concentration as this comes with certain assumptions. Rather, the signal is used to follow the dynamics of the hemoglobin concentration and thereby the CBF in the illumined portion of the cortex. NIRS cannot be used to examine deeper parts of the brain.^86^

### 2.7 Data Analysis

Spectral analysis is needed to convert the raw NIRS signal into oscillation parameters in the frequency domain. Only linear models will be considered here, though nonlinear models have shown comparable results.^87^ There are two common ways of doing the spectral analysis of the NIRS signals: the Fourier transform and the wavelet transform.

Time-domain analyses such as cerebral oximetry (Cox) index,^6^ mean velocity (Mx) index,^88^ and tissue oxygenation (Tox) index ^89^ will not be discussed in this paper as these analyses differ too greatly from frequency-domain analysis to be compared directly, despite being correlated to a certain degree.^90^

#### 2.7.1 Fourier transform

The Fourier transform is commonly used to decompose a function of time, i.e., the NIRS signal, into a frequency domain. There are many different variations of the Fourier transform. We will address a couple of them here, but will not be discussing them further in this paper. One of the variations can transform the NIRS signals in the time domain, but this will not account for specific frequency intervals, and therefore, is not suitable for analyzing LFOs and VLFOs. The frequency domain analysis generates certain frequency intervals, in which the oscillations occur and the amplitudes of the oscillations in these frequency intervals. A variation of this that is often used is called the fast Fourier transform. It generates a measure called power spectral density (PSD), which is the amplitude within a given signal in relation to the frequency of the signal. The PSD and the amplitude do not, however, say anything about the synchronicity of the oscillations.

#### 2.7.2 Transfer function analysis

The transfer function analysis (TFA) based on the Fourier transform is used to detect synchronization of LFOs and VFLOs and the method has been passed on from the TCD research paradigm.^60^^,^^78^^,^^91^ It is a black box input–output analysis, generating ratios of the input signal that is transferred to the output signal. In this context, the input can either be the ABP, mean arterial pressure, or $V_{\mathrm{MCA}}$, and the output being the NIRS signals. The analysis produces two certain measures, gain and phase shift. The gain is the amplitude of the input oscillations relative to the amplitude of the output NIRS oscillations. Equal amplitudes would, therefore, generate a gain of 1. The phase shift (or phase angle) is a measure of synchronization and if the oscillations occur at the exact same time the phase shift would be 0 deg. If phase shift is negative, the input oscillations occur before the output oscillations and vice versa, while a counterphase relationship would generate a phase shift of 180 deg.

The input in a TFA can also be the NIRS signal of a healthy hemisphere, which might serve as the best comparison if the individual subjects have a healthy hemisphere.

#### 2.7.3 Wavelet transform

The wavelet transform differs from the Fourier in that it transforms data into a time-frequency domain, whereas Fourier can only transform data into either frequency or time domain one at a time. This gives the transform some slight mathematical benefits and some other possibilities. The downside is a trade-off between spectral and temporal resolution. The wavelet transform can, therefore, generate the amplitude of examined oscillations, but also an instantaneous phase.^92^

In the NIRS oscillation research, the most commonly used concepts are wavelet coherence (WCO) and wavelet phase coherence (WPCO). WCO is a coherence determination of both the amplitude and the relative phase shift at once. The WCO is difficult to understand in the context of LFOs and VLFOs as it is hard to dissociate the importance the amplitude and the phase shift to the WCO. Instead, WPCO determines how constant the phase difference is between to signals in a certain frequency range. A WPCO of 1 means that the phase difference between two oscillations constant over the entire time series, whereas a WPCO of 0 would only be generated from two totally independent oscillating signals.

Amplitude and phase of Fourier and wavelet transform can be compared without conversion unlike synchronization analysis (phase shift and WPCO).^93^ To our knowledge, the only studies using the two methods on the same group of patients are conducted by Tachtsidis et al.^24^ and Rowley et al.^55^ showing similar trends in PSD, while it is difficult to assess it quantitatively from the publicized results. Other methods are described thoroughly and compared in an excellent review by Thewissen et al.^94^ regarding NIRS investigations of CA in neonates.

## 3 Methods

### 3.1 Search Strategy

A systematic search of literature was conducted in MEDLINE database through May 1, 2018. The search strategy was a combination of the NIRS and oscillations concepts. Broad terms were used to minimize the risk of missing any articles. The full search strategy is depicted in Table 2.

**Table 2:** Search strategy.

AND				
NIRS concept	Oscillations concept			Filters
“Spectroscopy, NIR” [MeSH] OR NIRS OR “NIR spectroscopy” OR “Cerebral oximetry” OR “Cerebral oxygenation” OR “Cerebral oxygen saturation”	Oscillation(s) OR Fluctuation(s) OR “M-wave(s)” OR M-wave(s) OR Vasomotion OR LFO(s) OR VLFO(s)	OR	Fourier OR Wavelet OR “Transfer function” OR Transform AND Amplitude(s) OR Gain OR “PSD” OR PSD OR Phase(s) OR Phase shift OR WPCO	Humans [MeSH terms] AND Adults [MeSH terms] AND English language [MeSH terms]

The search resulted in 176 potentially relevant articles. In addition, the identified articles’ reference lists were reviewed to detect any relevant missing articles. This search resulted in two additional articles. All 178 potentially relevant articles were screened for relevance according to the following criteria.

### 3.2 Inclusion and Exclusion Criteria

**Fig. 4:**
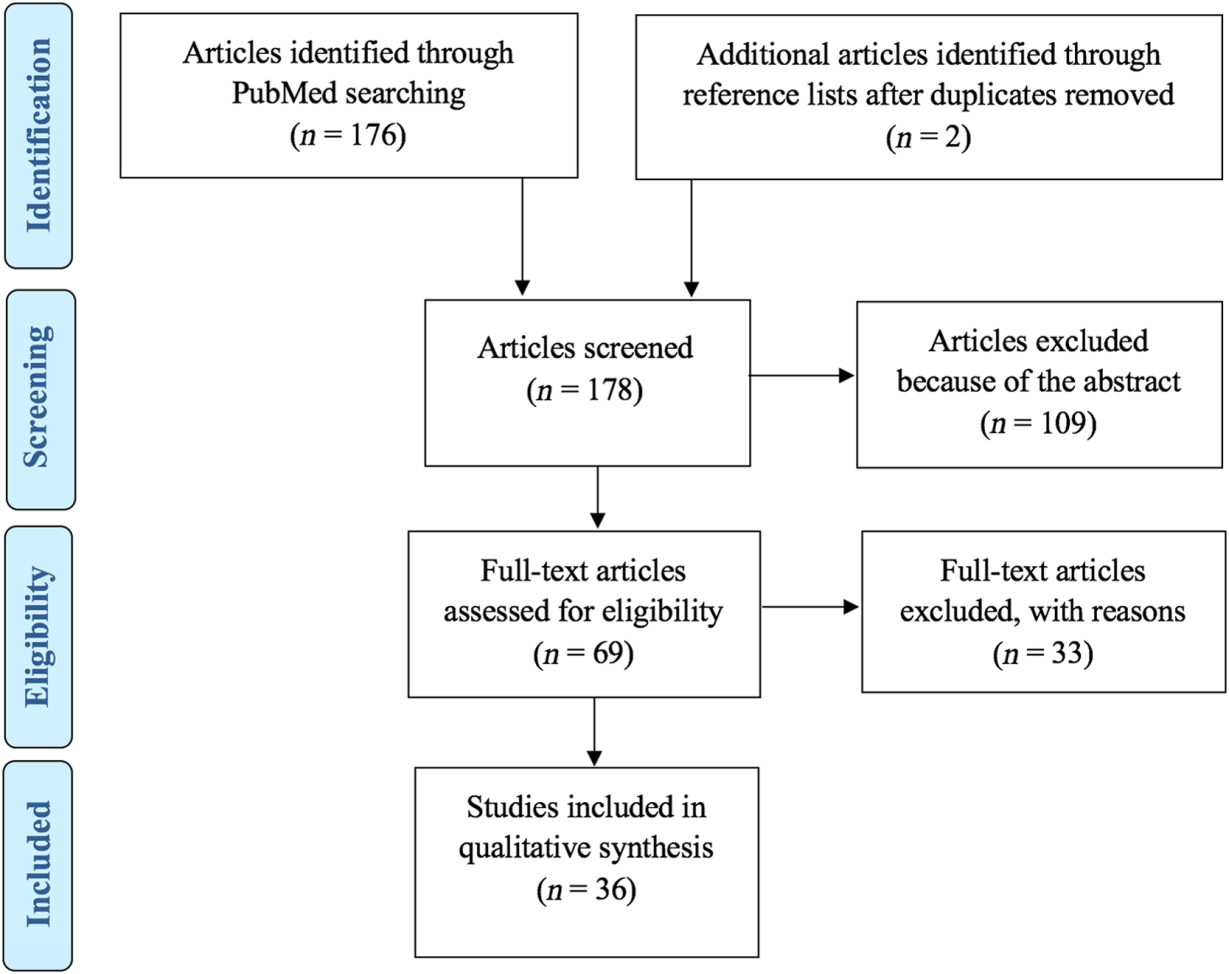
PRISMA flow diagram.

To be included, studies had to use NIRS to examine cerebral LFOs or VLFOs with frequency domain analysis in cerebrovascular diseases or related conditions preceding them including aging. Studies of healthy populations were also included as an indicator of normal variations, though articles focusing on technical or analytical aspects were excluded. Traumatic brain injury patients and intraoperative monitoring were excluded. Only original peer-reviewed studies presenting own results were included leading to the exclusion of reviews. The process is depicted in Fig. 4.

## 4 Results and Discussion

Thirty-six studies were included and reviewed. Studies were divided into four categories based on study population: healthy subjects, cerebral diseases with structural brain damage, symptomatic cerebrovascular disease with increased risk of further cerebrovascular damage, and asymptomatic populations with increased risk of cerebrovascular disease. Some studies overlapped these categories.

In the following, only OxyHb data will be included in the discussion. The DeoxyHb data conceivably have interesting findings, especially in functional activation studies and when comparing to BOLD-MRI measurements. However, the observed DeoxyHb-changes are mainly a reflection of the venous compartments, whereas OxyHb represents all vascular compartments.^95^ Additionally, the dynamic changes in OxyHb are closer related to the vascular compartments where CA is most active,^6^^,^^96^ and the OxyHb signals are more robust and also more reliable in the topical frequency spectrum^97^ making it the most suitable for evaluating CA. The transform parameters have been discussed previously.

Due to the heterogeneity of technical and analytical methods, no quantitative measures are listed in this section. Rather, results are presented as comparative measures as all studies in this area of research use some sort of control group. Most studies had a healthy control group, while others compared different stages of a disease or one hemisphere to the contralateral.

### 4.1 Healthy Subjects

**Fig. 5:**
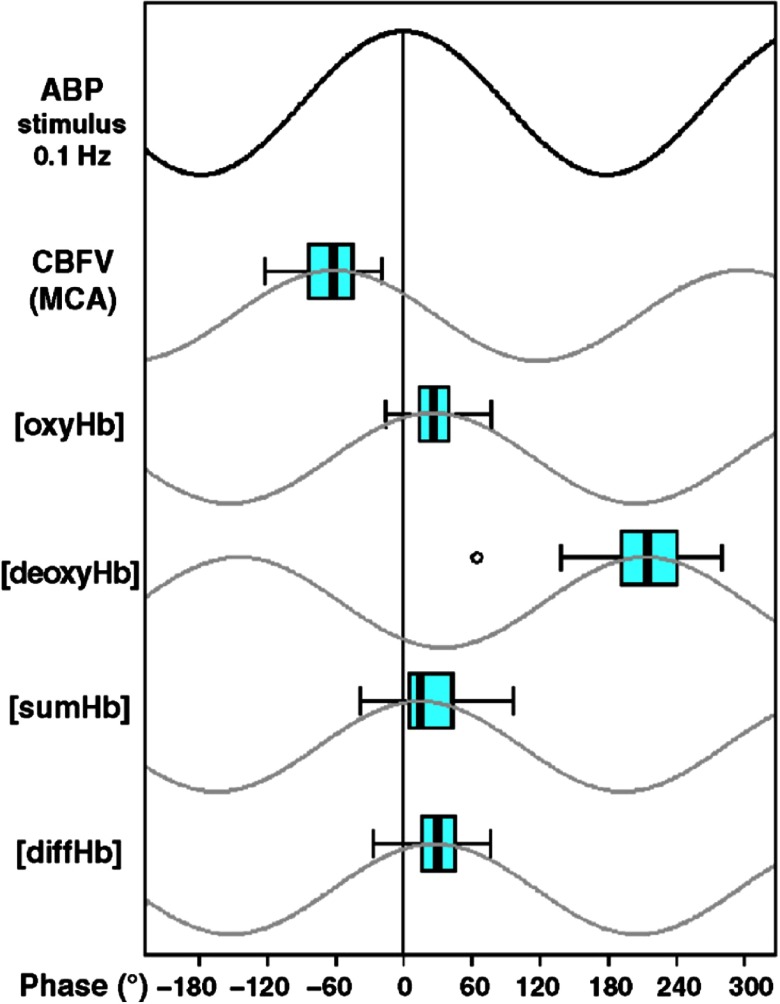
Physiological phase relationship between oscillations in ABP and different cerebral hemodynamic parameters. Schematic illustration of oscillations. SumHb=OxyHb+DeoxyHb. DiffHb=OxyHb−DeoxyHb. With permission from Reinhard et al.^98^

In a seminal study, Reinhard et al.^98^ measured LFOs in ABP, $V_{\mathrm{MCA}}$ and OxyHb in a healthy elderly population. The $V_{\mathrm{MCA}}$ LFOs preceded the ABP LFOs, which were again slightly ahead of OxyHb LFOs. All LFOs were highly correlated with each other and so the phase shifts merely represent a temporal lag between which vessels the oscillations are measured from Fig. 5. This finding was later confirmed in healthy young people by Philip et al.,^99^ who additionally showed equal phase shifts across gender, between left and right hemispheres, and over time. Neither visual,^80^ respiratory,^99^^,^^100^ motoric,^101^ nor positional stimulations^24^ have caused any significant changes in the synchronization of LFOs. However, desynchronization has been shown with poor sleep quality in healthy elderly^102^ and with sleep deprivation in healthy young people.^103^

In contrast, the amplitude of OxyHb LFOs is sensitive to stimulations and increases with sympathetic stimulations,^24^^,^^100^ deep breathing,^100^ and poor sleep,^102^ diminishes with hypercapnia,^80^ while visual stimulations did not affect them. No difference has been observed across gender or between hemispheres, but the day-to-day amplitude ratio seems to fluctuate considerably.^99^

Very little corresponding knowledge has been obtained about the normal variance of OxyHb VLFOs.

### 4.2 Cerebral Diseases with Structural Brain Damage

The studies in Table 3 were identified.

**Table 3:** Included studies of cerebral diseases with structural brain damage.

Authors	Population	Signals	Oscillations	Frequency range (Hz)	Analyses
Phillip et al.^105^	Acute stroke	OxyHb	LFO	0.09 to 0.11	Amplitude and synchronization
Li et al.^104^	Chronic stroke	OxyHb	LFO	0.04 to 0.15	Amplitude
			Upper VLFO	0.02 to 0.04	
Han et al.^112^	Chronic stroke	OxyHb	LFO	0.052 to 0.145	Synchronization
			Upper VLFO	0.021 to 0.052	
Han et al.^113^	Chronic stroke	OxyHb	LFO	0.052 to 0.145	Synchronization
			Upper VLFO	0.021 to 0.052	
Tan et al.^114^	Chronic stroke	OxyHb	LFO	0.052 to 0.145	Synchronization
			Upper VLFO	0.021 to 0.052	
			Bottom VLFO	0.0095 to 0.021	
Schroeter et al.^107^	CMA	OxyHb	LFO	0.07 to 0.12	Amplitude
			Upper VLFO	0.01 to 0.05	
van Beek et al.^108^	AD	$V_{\mathrm{MCA}}$	LFO	0.07 to 0.13	Amplitude and synchronization
		OxyHb	Upper VLFO	0.02 to 0.07	

#### 4.2.1 Amplitude

In ischemic stroke occurring more than 12 months ago, the amplitude of both LFOs and VLFOs was shown to be lower than in healthy age-matched controls.^104^ Phillip et al.^105^ investigated LFOs of acute ischemic strokes within 5 days of ictus, but did not examine a healthy control group. Thrombolysed patients, conceivably having suffered less damage to their brain, exhibited amplitudes equal to that of nonthrombolysed patients. The absolute amplitude ratio was borderline different between the two group and amplitude did not correlate with NIHSS on admission. According to the prevailing oscillation theories, decreased amplitudes would indicate a lower mean SNA or less myogenic (LFO) as well as neurogenic and metabolic (VLFO) autoregulatory activity. The former does not correlate with the common understanding of the autonomic nervous system (ANS) after stroke, where the SNS is believed to be overactive and dominating over the relatively inert parasympathetic nervous system.^106^ However, the smooth muscle loses its tone after a stroke, which is an effect known as vasomotor paralysis.^1^ The ability to contract is a prerequisite for the SNS to raise LFO amplitude not being met due to the stroke. The decline in myogenic CA activity is an indication of the vasomotor paralysis rendering the brain vulnerable to further damage and could be permanent as it can be observed at least 12 months at minimum after a stroke.

Schroeter et al.^107^ found a decreased LFO amplitude in patients with cerebral microangiopathy (CMA), though only due to hypertension. The amplitude of VLFOs in this study increased with visual stimulation supporting the theory of VLFO amplitude as in indication of possible metabolic control mechanism (though the applied range was an aggregate of metabolic and neurogenic VLFO frequencies). As CMA patients have damaged small vessels, one would have expected altered VLFO amplitude compared to the control group as the metabolic control occurs in the small vessels, but this was not the case possibly due to the aggregate VLFO range applied.

Newly diagnosed Alzheimer’s (AD) patients exhibited a higher LFO amplitude at rest in a study by van Beek et al.^108^ The increased myogenic activity could be explained by a higher mean SNA level as shown in studies of the ANS in AD.^109^^–^^111^ The difference in LFO amplitude vanished when the groups performed a repetitive sit–stand maneuver possibly because of the SNS responding to large ABP-changes in healthy controls. VLFO amplitude was equivalent in the two groups under both conditions expressing equal neurogenic and metabolic CA activity.

A TFA of these patients showed that gain between $V_{\mathrm{MCA}}$ and OxyHb VLFOs was increased compared to controls, which authors proposed as an indicator of reduced metabolic reserve or a reduced diffusion of oxygen as observed in positron emission tomography studies of AD. The LFO $V_{\mathrm{MCA}}$-OxyHb gain amplitude was similar across the two groups.

#### 4.2.2 Synchronization

Several studies have explored the LFOs in resting patients with a chronic cerebral infarction and all found a lower interhemispheric WPCO in stroke patients suggesting a lesser stability of the phase difference between the hemispheres compared to healthy people.^112^^–^^114^ This effect was interpreted as a loss of the control on myogenic CA activity in the smooth muscle of resistance vessels. Such a desynchronization was also seen in acute stroke as evidenced by a higher absolute interhemispheric phase shift.^105^ Interestingly, the interhemispheric phase shift proved to be well correlated to the National Institutes of Health Stroke Scale (NIHSS) and so the impaired CA was due to stroke size and not to treatment (thrombolysis or not).

VLFOs in the upper range exhibited lower interhemispheric WPCO in chronic stroke than control groups in two studies,^112^^,^^114^ while another showed an equal WPCO though with a smaller sample size.^113^ Lower WPCO would indicate a disruption of spontaneous neurogenic CA activity. Tan et al.^114^ also inspected the oscillations in the bottom VLFO range, which also had a lower WPCO and thus a reduced synchronicity of the metabolic control mechanism.

AD patients displayed a higher $V_{\mathrm{MCA}}$-OxyHb phase shift in the VLFO range and authors explained this as differences in either active regulation mechanisms or in passive properties in the cerebral vasculature.^108^ They also found an equal phase shift in the LFO range suggesting intact coordination of myogenic CA.

### 4.3 Symptomatic Diseases with Increased Risk of Further Damage

The studies in Table 4 were identified.

**Table 4:** Included studies of symptomatic diseases with increased risk of further damage.

Authors	Population	Signals	Oscillations	Frequency range (Hz)	Analyses
Reinhard et al. ^98^	Unilateral CS	ABP	LFO	≈0.1	Synchronization
		$V_{\mathrm{MCA}}$			
		OxyHb			
Phillip et al. ^115^	Symptomatic CS	ABP	LFO	0.09 to 0.11	Amplitude and synchronization
		OxyHb			
Reinhard et al.^124^	Unilateral CS	ABP	LFO	0.095 to 0.105	Synchronization
		OxyHb			
Oldag et al. ^126^	Severe unilateral MCA stenosis	ABP	LFO	0.08 to 0.12	Synchronization
		OxyHb			
He et al. ^117^	Moyamoya	OxyHb	LFO	0.06 to 0.15	Amplitude
			Upper VLFO	0.02 to 0.06	
			Bottom VLFO	0.0095 to 0.02	
Schytz et al. ^119^	FHM ± common migraine	OxyHb	LFO	0.09 to 0.11	Amplitude
Schytz et al.^120^	OSA	OxyHb	LFO	0.05 to 0.15	Amplitude
Kolyva et al.^122^	Malaria	OxyHb	LFO	0.04 to 0.15	Amplitude
			Upper VLFO	0.02 to 0.04	

Studies of migraine, obstructive sleep apnea (OSA), and malaria were included in the review, though the risk association to cerebrovascular disease is not strong enough to draw any definite conclusions. However, these diseases have cerebrovascular traits that make them interesting in this context.

#### 4.3.1 Amplitude

Symptomatic carotid stenosis (CS) patients were examined by Phillip et al.,^115^ which revealed equal gain between ABP and OxyHb LFOs when comparing to both the contralateral hemisphere and the hemispheres of healthy controls. However, an interhemispheric TFA was performed and showed a higher amplitude ratio due to lower amplitude on the hypoperfusion side. This indicates lower myogenic activity in the microvasculature distal to the stenosis as an expression of compensatory dilation due to inadequate perfusion and oxygen delivery.

Moyamoya disease is characterized by intimal proliferation and stenosis of both the internal carotid arteries as well as its intracranial branches thereby creating the need for collateral angiogenesis looking like a dust cloud on angiography.^116^ The disease was inspected with NIRS, which showed lower amplitude in the LFO range and bottom VFLO range.^117^ In accordance with histological studies proving smooth muscle degeneration as the intima grows expansively,^118^ this result points to a decrease in spontaneous smooth muscle activity.

Schytz et al.^119^ examined both patients with familial hemiplegic migraine (FHM), common migraine, and OSA.^120^ Patients suffering from FHM combined with common migraine had induced attacks with infusion of glyceryl trinitrate, and this was accompanied by higher LFO amplitude possibly caused by a reflectory increase in SNA and smooth muscle tone. OSA patients were examined before and 2 months after initiation of continuous positive airway pressure (CPAP) treatment. Oddly, OSA patients did not exhibit any difference to controls in LFO amplitude before CPAP despite OSA usually raising the general sympathetic tone^121^ and thus the smooth muscle activity, but perhaps this was disrupted by endothelial damage also associated with OSA^120^. However, they displayed a significant decline after treatment, reflecting the expected modulation of sympathetic activity.

In malaria patients, no difference in LFO amplitude was detected between cerebral and noncerebral malaria.^122^ Upper VLFO amplitude, however, was lower in cerebral malaria perhaps demonstrating the changes in microvasculature flow prompted by the parasite.^123^ Authors state an increase in VLFO amplitude after recovery, but with no mention of which patients were included in the follow-up analysis.

#### 4.3.2 Synchronization

CS has been under scrutiny by TFA due to the inherent nature of the disease. Reinhard et al.^98^ were first to investigate it with NIRS as the output measure. Compared to both healthy controls and the contralateral hemisphere, respiratory amplified LFOs in $V_{\mathrm{MCA}}$ and OxyHb were delayed relative to ABP LFOs, but only the phase shift to the former was statistically significant. The normal counterphase relationship of OxyHb–DeoxyHb was also abrogated. These changes point to a desynchronization and thus impaired CA. Results from such patients must be carefully treated, as it is difficult to dissociate the effect of CS from other risk factors and infarction, though only a minor portion had any symptoms in this study. Authors later solidified their findings in an examination using a multichannel NIRS system, which also showed CA being mostly impaired in the MCA/ACA (anterior cerebral artery) border zone.^124^

Similarly, Philip et al.^115^ arrived at a borderline altered phase shift when comparing to the contralateral hemisphere in resting symptomatic CS patients also demonstrating an impaired CA, but no difference compared to healthy controls. Using interhemispheric OxyHb TFA, authors presented an altered absolute interhemispheric phase shift with borderline significance. Interhemispheric measures seem more intuitively correct as it would minimize anatomical variations^125^ despite the noteworthy limitation of needing a healthy contralateral hemisphere to compare with.

The trend also occurs in severe unilateral MCA stenosis although the examined patients were asymptomatic. Increased LFO ABP-OxyHb phase shift was observed in the affected hemisphere over the entire hemisphere and even higher in the core MCA distribution area.^126^ A subgroup was examined for CVR, which showed that diminished CVR led to higher phase shifts. The desynchronization of myogenic CA could thus be the consequence of the downstream vessels not being able to compensate for the MCA stenosis.

### 4.4 Asymptomatic Conditions with Increased Risk of Cerebrovascular Disease

The studies in Table 5 were identified.

**Table 5:** Included studies of asymptomatic conditions with increased risk of further damage.

Author	Population	Signals	Oscillations	Frequency range (Hz)	Analyses
Li et al.^128^	Risk of stroke − elevated $V_{\mathrm{MCA}}$	OxyHb	LFO	0.06 to 0.15	Amplitude
			Upper VLFO	0.02 to 0.06	
			Bottom VLFO	0.005 to 0.02	
Li et al.^129^	Hypertension ± elevated $V_{\mathrm{MCA}}$	OxyHb	LFO	0.06 to 0.15	Amplitude
			Upper VLFO	0.02 to 0.06	
			Bottom VLFO	0.005 to 0.02	
Li et al.^138^	Hypertension	OxyHb	LFO	0.05 to 0.15	Synchronization
			Upper VLFO	0.02 to 0.05	
Zeller et al.^130^	Elderly ± MCI	OxyHb	LFO	0.07 to 0.11	Amplitude
Schroeter et al.^131^	Elderly	OxyHb	LFO	0.07 to 0.11	Amplitude
			Upper VLFO	0.01 to 0.05	
Peng et al.^136^	Elderly	HR	LFO	0.0625 to 0.125	Amplitude and synchronization
		ABP			
		$V_{\mathrm{MCA}}$			
		OxyHb			
Philip et al.^99^	Elderly	ABP	LFO	0.09 to 0.11	Amplitude and synchronization
		OxyHb			
Li et al.^132^	Elderly	OxyHb	LFO	0.06 to 0.15	Amplitude
			Upper VLFO	0.02 to 0.06	
			Bottom VLFO	0.0095 to 0.02	
Vermeij et al.^135^	Elderly	ABP	LFO	0.07 to 0.2	Amplitude and synchronization
		OxyHb	Upper VLFO	0.02 to 0.07	
Oudegeest-Sander et al.^137^	Elderly	$V_{\mathrm{MCA}}$	LFO	0.07 to 0.2	Amplitude and synchronization
		OxyHb	Upper VLFO	0.02 to 0.07	
Cui et al.^140^	Elderly	ABP	LFO	0.05 to 0.15	Synchronization
		OxyHb	Upper VLFO	0.02 to 0.05	
			Bottom VLFO	0.0095 to 0.02	
Gao et al.^139^	Elderly	ABP	LFO	0.05 to 0.15	Synchronization
		OxyHb	Upper VLFO	0.02 to 0.05	
			Bottom VLFO	0.0095 to 0.02	
Song et al.^133^	Elderly	OxyHb	LFO	0.06 to 0.15	Amplitude
			Upper VLFO	0.02 to 0.06	
			Bottom VLFO	0.01 to 0.02	
Tan et al. ^134^	Elderly	OxyHb	LFO	0.052 to 0.145	Amplitude and synchronization
			Upper VLFO	0.021 to 0.052	
			Bottom VLFO	0.0095 to 0.021	
Wang et al.^141^	Elderly	OxyHb	LFO	0.052 to 0.145	Synchronization
			Upper VLFO	0.021 to 0.052	
			Bottom VLFO	0.0095 to 0.021	

#### 4.4.1 Amplitude

Elevated $V_{\mathrm{MCA}}$ has been recognized as a measure of intracerebral atherosclerosis, and therefore, a risk factor for stroke.^127^ People with this condition displayed lower amplitude in LFO, upper and bottom VFLO ranges, though the former two were borderline significant.^128^ This provides indication of decreases in myogenic, neurogenic, and metabolic activity of the cerebral vasculature with elevated $V_{\mathrm{MCA}}$, which could all be attributed to the stiffening of vessel walls in atherosclerosis.

An important factor in the development of atherosclerosis is arterial hypertension, in which Li et al.^129^ examined with half of the hypertension subjects also exhibiting elevated $V_{\mathrm{MCA}}$. Hypertension increased the LFO amplitude as the result of myogenic autoregulatory mechanisms being activated to protect the brain in conform to general CA knowledge.^1^ The group with elevated $V_{\mathrm{MCA}}$ had amplitudes between that of the hypertension group and the healthy controls. This dampening of amplitude consolidates the findings in the former study of elevated $V_{\mathrm{MCA}}$.^128^ Noteworthy is the opposite trend of LFO amplitude in CMA patients as the result of hypertension^107^ possibly elucidating that CMA patients do not have the ability to increase their myogenic CA in response to the challenge of hypertension. VLFO amplitudes did not show any definite trends in hypertension.

LFO amplitude has been shown to decrease with age in numerous studies with subjects both at rest^130^^,^^131^^,^^132^^,^^133^^,^^134^ and with stimulations of visual,^131^ cognitive,^135^ and positional character.^136^^,^^133^ Generally, this has been interpreted as an expression of vessel stiffening with age, and therefore, less microvascular smooth muscle activity. This trend was even more pronounced in elderly with mild cognitive impairment (MCI), though only in the parietal lobes.^130^ TFA showed no difference in LFO ABP-OxyHb gain across age groups during rest^99^^,^^135^ or cognitive memory task.^135^

VLFO amplitude in aging exhibited a similar decline^132^ although more apparent with stimulations^131^^,^^135^^,^^133^ displaying an age effect on neurogenic and metabolic CA. TFA of ABP-OxyHb VLFO during the cognitive test by Vermeij et al.^135^ showed no effect of age or cognitive load on gain.

During sit–stand maneuvers performed in a study by Oudegeest-Sander et al.,^137^ authors found no difference in $V_{\mathrm{MCA}}$-OxyHb gain across age groups, but a trend toward higher gain was conveyed in their regression analysis. Reduced distensibility and thereby less damping of $V_{\mathrm{MCA}}$ oscillations in the elderly were proposed to account for this difference.

#### 4.4.2 Synchronization

The interhemispheric WPCO approach was applied to hypertension patients and resulted in lower LFO WPCO and equal WPCO in the upper VLFO range.^138^ This loss of synchronicity was connected to a reduced control of the microvascular smooth muscle activity. The stroke study by Han et al.^112^ examined a subpopulation also suffering from hypertension and found an even lower WPCO in the upper VLFO range suggesting additional desynchronization of neurogenic CA activity, while hypertension did not desynchronize LFOs any further.

The synchronization of oscillations in the aging brain has been well examined. Peng et al.^136^ used a synchronicity analysis called wavelet cross correlation (WCC, higher values indicating stronger synchronicity). It showed that the WCC for HR-OxyHb, ABP-OxyHb, and $V_{\mathrm{MCA}}$-OxyHb was equal in the LFO range when subjects remained at supine rest but increased substantially more in young people with head-up tilt test and with active standing in another study.^139^ This implies an increase in synchronization between systemic and CBF under sympathetic challenges such as positional changes and that this mechanism is impaired in the elderly.

ABP-OxyHb LFO phase analyses have disclosed equal phase shifts in young and elderly under rest^99^^,^^135^ and during cognitive testing.^135^ Wavelet studies have come to the same conclusion, as the ABP-OxyHb WPCO was homogenous across age groups at rest.^140^^,^^139^ Meanwhile, interhemispheric analyses found lower WPCO with aging indicating desynchronization of myogenic CA at rest.^134^^,^^141^

Oudegeest-Sander et al.^137^ examined the VLFOs during a sit-stand maneuver and their regression analysis showed a trend toward higher $V_{\mathrm{MCA}}$-OxyHb phase shift with age explained by increased vascular tortuosity. The WPCO between VLFOs of ABP and OxyHb in the elderly has also been investigated. At rest synchronization of neurogenic CA activity was higher in the elderly perhaps to compensate for the decline in myogenic CA activity.^140^^,^^139^ During active standing, this difference evened out and both the neurogenic and metabolic CA synchronization was equivalent to the young.^139^ Interhemispheric WPCO analysis also showed intact synchronization of metabolic and neurogenic activity.^134^

## 5 Limitations

Before making any definitive conclusions based on the reviewed material, several limitations must be acknowledged. The penetration depth of infrared light prevents NIRS from ever examining deeper parts of the brain than the available cortex and can never be used to determine whether the observations are reflecting global hemispheric trends or local phenomena despite the use of multichannel systems. In general, the reviewed examinations have applied an interoptode distance of 3 to 5 cm to get the deepest penetration possible, but with no coherency across studies. Although signals from the skin and skull could pollute the cortical signal, the method of short separation optodes rectifies this shortcoming in most cases.

NIRS assumes that the infrared light passes through tissue with a constant spread and that only hemoglobin absorbs the light, which are both reasonable. The emanating signal is comprised of the entire tissue and all the different vascular compartments, which is hard to separate and only recently made conceivable with NIRS.^142^ The infrared light in the spectrum utilized has a wavelength between 650 and 950 nm, but there is no unified agreement, in which exact wavelengths are most appropriate. Because the relative absorption of OxyHb and DeoxyHb changes with the wavelength, there is a difference between the measured chromophores and thus the examined vascular compartment when using different wavelengths.^95^^,^^143^ NIRS also assumes that the measured hemoglobin concentration is the homogenous in the illuminated tissue, which is not necessarily the case, especially when local pathology is involved.

Processing NIRS signals can be done in a variety of ways to remove motion artefacts and while every process has its advantages, none have proved to be superior to others. The technique is more sensitive to motion artefacts than it is credited for, which will either limit the possible functional stimulations or raise the requirements for postprocessing.

The spectral analysis of NIRS signals can either be performed with Fourier or wavelet transforms and while the two methods are mathematically equivalent, the outcomes in the synchronization analysis cannot be compared directly. Also the differences in technical setup, postprocessing, and spectral analyses render any sort of metaanalysis impossible.

The oscillation analysis method is quite demanding in several ways. The nature of LFOs requires rather long recordings to build a significant data foundation as LFOs only occur 6 times per minute and VLFOs 1 to 3 times per minute. The analysis itself is quite complex and commands skilled personnel and time. Also, as shown in Tables 3–5, there is a need for coherency in the applied frequency ranges. These factors constitute a substantial obstacle of monitoring for immediate changes in CA and thus the clinical implementation.

In this paper, both spontaneous oscillations and oscillations enhanced with stimulations of appropriate frequency have been included. Debate remains between scientists as to which method is better.^144^ While spontaneous oscillations are claimed to have higher signal-to-noise ratio, this could be accounted for in the analysis and provide a reasonable method for evaluating CA in patients where stimulations can be challenging to perform, e.g., stroke or other cerebrovascular diseases.

Average population size in NIRS oscillation studies is not particularly large and the need for larger-scale investigations is obvious in order to solidify the current knowledge and showcase the possible clinical utilization. However, the technique suffers somewhat due to the perpetual technical and analytical development quickly causing results to be viewed as either outdated or inappropriate for comparison with other studies. It also makes the continuous examination of healthy control groups imperative. There have not yet been established any quantitative measure of intact or impaired CA, which also leaves power calculations impossible.

Caution should be advised in the oscillation research, as investigation of too many oscillating chromophores and NIRS parameters could potentially be more misleading than progressive. The absence of focus in both study design and outcome is dangerous when dealing with relatively small sample sizes. Additionally, the term “altered oscillations” is often applied in discussions of results across different parameters and should be avoided. Each parameter should be carefully analyzed separately as the current study has attempted.

More research is clearly needed both in experimental and clinical studies to improve our knowledge of the oscillations in general and expand the pathophysiological understanding of cerebrovascular diseases and conditions that lead to them. The method would benefit greatly from more coherent technical and analytical procedures, while advances in these areas could also provide the basis for implementation in clinical settings.

## 6 Conclusion

CA is a complex phenomenon ensuring adequate perfusion to the brain by myogenic, neural, metabolic, and possibly other mechanisms. The modulation of systemic oscillations in the low- and very low-frequency spectrum measured in cerebral vessels is thought to be an expression of the exerted autoregulation. Near-infrared spectroscopy can be used to examine regional cerebral perfusion and thus evaluate CA in cerebrovascular diseases and related conditions. The outcome of LFO amplitude is relatively robust in quantifying the myogenic CA from the smooth muscle cells in the microvasculature, which is lower in stroke, atherosclerosis, and with aging, but must be interpreted with great care as it can be affected by sympathetic activity as in hypertension. LFO synchronization analyses of oscillations have shown disruption of normally well-coordinated autoregulatory actions in stroke, internal carotid, and MCA stenosis as well as in hypertension in accordance with other measurement techniques, but the optimal comparison remains uncertain. VLFO amplitudes suggest lower metabolic and neurogenic CA in stroke, atherosclerosis, and aging, but are generally not as consistent as LFO amplitude, in part due to incoherent frequency ranges. VLFOs are desynchronized in stroke, intact in hypertension, and with aging. Although the outcomes can never stand alone, they seem able to enhance our knowledge of CA. Despite certain limitations, the oscillation analysis of NIRS data could be a valuable tool to both researchers and physicians in the future. Additional research and more coherent methodologies are needed.

## References

[1] Paulson O. B. Strandgaard S. Edvinsson L., “Cerebral autoregulation,” Cerebrovasc. Brain Metab. Rev. 2(2), 161–92 (1990). CEMREV 1040-8827 2201348

[2] Donnelly J. Aries M. J. Czosnyka M., “Further understanding of cerebral autoregulation at the bedside: possible implications for future therapy,” Expert Rev. Neurother. 15, 169–185 (2015). 10.1586/14737175.2015.996552 25614952

[3] Lassen N. A., “Cerebral blood flow and oxygen consumption in man,” Physiol. Rev. 39, 183–238 (1959). PHREA7 0031-9333 10.1152/physrev.1959.39.2.183 13645234

[4] Faraci F. M. Heistad D. D., “Regulation of large cerebral arteries and cerebral microvascular pressure,” Circ. Res. 66, 8–17 (1990). 10.1161/01.RES.66.1.8 2403863

[5] Moerman A. De Hert S., “Recent advances in cerebral oximetry. Assessment of cerebral autoregulation with near-infrared spectroscopy: myth or reality?” F1000Res. 6, 1615 (2017). 10.12688/f1000research 29026526 PMC5583743

[6] Brady K. M. et al., “Continuous time-domain analysis of cerebrovascular autoregulation using near-infrared spectroscopy,” Stroke 38, 2818–2825 (2007). SJCCA7 0039-2499 10.1161/STROKEAHA.107.485706 17761921 PMC2377358

[7] Chillon J. M. Baumbach G. L., “Autoregulation of cerebral blood flow,” in Cerebrovascular Diseases, 1st ed., Welch K. et al., Eds., pp. 51–54, Academic Press, San Diege, California (1997).

[8] Folino A. F., “Cerebral autoregulation and syncope,” Prog. Cardiovasc. Dis. 50, 49–80 (2007). PCVDAN 0033-0620 10.1016/j.pcad.2007.01.001 17631437

[9] Lassen N. A. Agnoli A., “The upper limit of autoregulation of cerebral blood flow on the pathogenesis of hypertensive encephalopathy,” Scand. J. Clin. Lab. Invest. 30, 113–116 (1972). SJCLAY 0036-5513 10.3109/00365517209081099 4640619

[10] Phillips S. J. Whisnant J. P., “Hypertension and the brain,” Arch. Intern. Med. 152, 938–945 (1992). AIMDAP 0003-9926 10.1001/archinte.1992.00400170028006 1580719

[11] Donnelly J. et al., “Regulation of the cerebral circulation: bedside assessment and clinical implications,” Crit. Care 20, 129 (2016). 10.1186/s13054-016-1293-6 27145751 PMC4857376

[r12] Folkow B., “Description of the myogenic hypothesis,” Circ. Res. 15(Suppl 1), 279–287 (1964). CIRUAL 0009-7330 14206315

[13] Peterson E. C. Wang Z. Britz G., “Regulation of cerebral blood flow,” Int. J. Vasc. Med. 2011, 1–8 (2011). 10.1155/2011/823525 PMC3144666 21808738

[14] Rubanyi G. M., “Endothelium-derived relaxing and contracting factors,” J. Cell Biochem. 46, 27–36 (1991). 10.1002/(ISSN)1097-4644 1874796

[15] Kontos H. A. Wei E. P., “Oxygen-dependent mechanisms in cerebral autoregulation,” Ann. Biomed. Eng. 13, 329–334 (1985). ABMECF 0090-6964 10.1007/BF02584251 4037462

[16] Aaslid R. et al., “Cerebral autoregulation dynamics in humans,” Stroke 20, 45–52 (1989). SJCCA7 0039-2499 10.1161/01.STR.20.1.45 2492126

[17] Hamner J. W. Tan C. O., “Relative contributions of sympathetic, cholinergic, and myogenic mechanisms to cerebral autoregulation,” Stroke 45, 1771–1777 (2014). SJCCA7 0039-2499 10.1161/STROKEAHA.114.005293 24723314 PMC4102642

[18] Seifert T. Secher N. H., “Sympathetic influence on cerebral blood flow and metabolism during exercise in humans,” Prog. Neurobiol. 95, 406–426 (2011). PGNBA5 0301-0082 10.1016/j.pneurobio.2011.09.008 21963551

[19] Meng L. Gelb A. W., “Regulation of cerebral autoregulation by carbon dioxide,” Anesthesiology 122, 196–205 (2015). ANESAV 0003-3022 10.1097/ALN.0000000000000506 25401418

[20] Gould I. G. et al., “The capillary bed offers the largest hemodynamic resistance to the cortical blood supply,” J. Cereb. Blood Flow Metab. 37, 52–68 (2017). 10.1177/0271678X16671146 27780904 PMC5363755

[21] Hall C. N. et al., “Capillary pericytes regulate cerebral blood flow in health and disease,” Nature 508, 55–60 (2014). 10.1038/nature13165 24670647 PMC3976267

[22] Mayer S., “Studien zur physiologie des herzens und der blutgefässe,” Sitz Kaiser Akad Wiss 74, 281–307 (1876).

[23] Julien C., “The enigma of Mayer waves: facts and models,” Cardiovasc. Res. 70, 12–21 (2006). CVREAU 0008-6363 10.1016/j.cardiores.2005.11.008 16360130

[24] Tachtsidis I. et al., “Investigation of cerebral haemodynamics by near-infrared spectroscopy in young healthy volunteers reveals posture-dependent spontaneous oscillations,” Physiol. Meas. 25, 437–445 (2004). PMEAE3 0967-3334 10.1088/0967-3334/25/2/003 15132309

[25] Grasso R. et al., “Arterial baroreceptors are not essential for low frequency oscillation of arterial pressure,” J. Auton. Nerv. Syst. 50, 323–331 (1995). JASYDS 0165-1838 10.1016/0165-1838(94)00103-Q 7714326

[r26] Montano N. et al., “Effects of spinal section and of positive-feedback excitatory reflex on sympathetic and heart rate variability,” Hypertension 36, 1029–1034 (2000). 10.1161/01.HYP.36.6.1029 11116120

[r27] Montano N. et al., “Presence of vasomotor and respiratory rhythms in the discharge of single medullary neurons involved in the regulation of cardiovascular system,” J. Auton. Nerv. Syst. 57, 116–122 (1996). JASYDS 0165-1838 10.1016/0165-1838(95)00113-1 8867094

[28] Preiss G. Polosa C., “Patterns of sympathetic neuron activity associated with Mayer waves,” Am. J. Physiol. 226, 724–730 (1974). AJPHAP 0002-9513 10.1152/ajplegacy.1974.226.3.724 4817426

[29] Guzzetti S. et al., “Influences of neural mechanisms on heart period and arterial pressure variabilities in quadriplegic patients,” Am. J. Physiol. 266, H1112–H1120 (1994). AJPHAP 0002-9513 10.1152/ajpcell.1994.266.4.C1112 8160814

[r30] Inoue K. et al., “Power spectral analysis of blood pressure variability in traumatic quadriplegic humans,” Am. J. Physiol. 260, H842–H847 (1991). AJPHAP 0002-9513 10.1152/ajpcell.1991.260.3.C658 2000979

[31] Koh J. et al., “Human autonomic rhythms: vagal cardiac mechanisms in tetraplegic subjects,” J. Physiol. 474, 483–495 (1994). JPHYA7 0022-3751 10.1113/jphysiol.1994.sp020039 8014908 PMC1160339

[32] Guyton A. C. Harris J. W., “Pressoreceptor-autonomic oscillation; a probable cause of vasomotor waves,” Am. J. Physiol. 165, 158–166 (1951). 10.1152/ajplegacy.1951.165.1.158 14829585

[33] Cerutti C. Barres C. Paultre C., “Baroreflex modulation of blood pressure and heart rate variabilities in rats: assessment by spectral analysis,” Am. J. Physiol. 266, H1993–H2000 (1994). AJPHAP 0002-9513 10.1152/ajpheart.1994.266.5.H1993 8203598

[r34] Jacob H. J. et al., “Spectral analysis of arterial pressure lability in rats with sinoaortic deafferentation,” Am. J. Physiol. 269, R1481–R1488 (1995). AJPHAP 0002-9513 10.1152/ajpregu.1995.269.6.R1481 8594953

[35] Julien C. et al., “Hemodynamic analysis of arterial pressure oscillations in conscious rats,” J. Auton. Nerv. Syst. 50, 239–252 (1995). JASYDS 0165-1838 10.1016/0165-1838(94)00095-2 7714320

[36] Barres C. de Souza Neto E. P. Julien C., “Effect of alpha-adrenoceptor blockade on the 0.4 Hz sympathetic rhythm in conscious rats,” Clin. Exp. Pharmacol. Physiol. 28, 983–985 (2001). 10.1046/j.1440-1681.2001.03561.x 11903298

[r37] Japundzic N. et al., “Spectral analysis of blood pressure and heart rate in conscious rats: effects of autonomic blockers,” J. Auton. Nerv. Syst. 30, 91–100 (1990). JASYDS 0165-1838 10.1016/0165-1838(90)90132-3 1973426

[38] Rubini R. et al., “Power spectrum analysis of cardiovascular variability monitored by telemetry in conscious unrestrained rats,” J. Auton. Nerv. Syst. 45, 181–190 (1993). JASYDS 0165-1838 10.1016/0165-1838(93)90050-5 8106708

[39] Cevese A. et al., “Baroreflex and oscillation of heart period at 0.1 Hz studied by alpha-blockade and cross-spectral analysis in healthy humans,” J. Physiol. 531, 235–244 (2001). JPHYA7 0022-3751 10.1111/tjp.2001.531.issue-1 11179406 PMC2278442

[40] van de Borne P. et al., “Contrasting effects of phentolamine and nitroprusside on neural and cardiovascular variability,” Am. J. Physiol. Heart Circ. Physiol. 281, H559–H565 (2001). 10.1152/ajpheart.2001.281.2.H559 11454557

[41] Kimmerly D. S. et al., “Circulating norepinephrine and cerebrovascular control in conscious humans,” Clin. Physiol. Funct. Imaging 23, 314–319 (2003). 10.1046/j.1475-0961.2003.00507.x 14617260

[42] Barres C. Cheng Y. Julien C., “Steady-state and dynamic responses of renal sympathetic nerve activity to air-jet stress in sinoaortic denervated rats,” Hypertension 43, 629–635 (2004). 10.1161/01.HYP.0000115384.01463.61 14732727

[r43] Bertram D. et al., “Differential responses of frequency components of renal sympathetic nerve activity to arterial pressure changes in conscious rats,” Am. J. Physiol. Regul. Integr. Comp. Physiol. 289, R1074–R1082 (2005).0363-6119 10.1152/ajpregu.00270.2005 15932970

[r44] Janssen B. J. et al., “Frequency-dependent modulation of renal blood flow by renal nerve activity in conscious rabbits,” Am. J. Physiol. 273, R597–R608 (1997). AJPHAP 0002-9513 10.1152/ajpregu.1997.273.2.R597 9277544

[45] Malpas S. C. et al., “Contribution of renal nerves to renal blood flow variability during hemorrhage,” Am. J. Physiol. 274, R1283–R1294 (1998). AJPHAP 0002-9513 10.1152/ajpcell.1998.274.5.C1283 9644041

[46] Castiglioni P. et al., “Mechanisms of blood pressure and heart rate variability: an insight from low-level paraplegia,” Am. J. Physiol. Regul. Integr. Comp. Physiol. 292, R1502–R1509 (2007).0363-6119 10.1152/ajpregu.00273.2006 17122332

[r47] Cooke W. H. et al., “Human responses to upright tilt: a window on central autonomic integration,” J. Physiol. 517(Pt 2), 617–628 (1999). JPHYA7 0022-3751 10.1111/tjp.1999.517.issue-2 10332107 PMC2269357

[r48] Furlan R. et al., “Oscillatory patterns in sympathetic neural discharge and cardiovascular variables during orthostatic stimulus,” Circulation 101, 886–892 (2000). CIRCAZ 0009-7322 10.1161/01.CIR.101.8.886 10694528

[r49] Lucini D. et al., “Hemodynamic and autonomic adjustments to real life stress conditions in humans,” Hypertension 39, 184–188 (2002). 10.1161/hy0102.100784 11799100

[50] Pagani M. et al., “Relationship between spectral components of cardiovascular variabilities and direct measures of muscle sympathetic nerve activity in humans,” Circulation 95, 1441–1448 (1997). CIRCAZ 0009-7322 10.1161/01.CIR.95.6.1441 9118511

[51] Taylor J. A. et al., “Low-frequency arterial pressure fluctuations do not reflect sympathetic outflow: gender and age differences,” Am. J. Physiol. 274, H1194–H1201 (1998). AJPHAP 0002-9513 9575922 10.1152/ajpheart.1998.274.4.H1194

[52] van de Borne P. et al., “Relationship between repeated measures of hemodynamics, muscle sympathetic nerve activity, and their spectral oscillations,” Circulation 96, 4326–4332 (1997). CIRCAZ 0009-7322 10.1161/01.CIR.96.12.4326 9416900

[53] Stefanovska A. Bracic M. Kvernmo H. D., “Wavelet analysis of oscillations in the peripheral blood circulation measured by laser Doppler technique,” IEEE Trans. Biomed. Eng. 46, 1230–1239 (1999). IEBEAX 0018-9294 10.1109/10.790500 10513128

[54] Intaglietta M., “Vasomotion and flowmotion: physiological mechanisms and clinical evidence,” Vasc. Med. 1, 101–112 (1990). VMEREI 10.1177/1358836x9000100202

[55] Rowley A. B. et al., “Synchronization between arterial blood pressure and cerebral oxyhaemoglobin concentration investigated by wavelet cross-correlation,” Physiol. Meas. 28, 161–173 (2007). PMEAE3 0967-3334 10.1088/0967-3334/28/2/005 17237588

[56] Harper A. M. et al., “The influence of sympathetic nervous activity on cerebral blood flow,” Arch. Neurol. 27, 1–6 (1972). 10.1001/archneur.1972.00490130003001 4626103

[57] Lundberg N., “Continuous recording and control of ventricular fluid pressure in neurosurgical practice,” Acta Psychiatr. Scand. Suppl. 36, 1–193 (1960). ASSUA6 0065-1591 13764297

[58] Mautner-Huppert D. et al., “B-waves in healthy persons,” Neurol. Res. 11, 194–196 (1989). 10.1080/01616412.1989.11739891 2576100

[59] Newell D. W. et al., “The relationship of blood flow velocity fluctuations to intracranial pressure B waves,” J. Neurosurg. 76, 415–421 (1992). JONSAC 0022-3085 10.3171/jns.1992.76.3.0415 1738020

[60] Giller C. A., “The frequency-dependent behavior of cerebral autoregulation,” Neurosurgery 27, 362–368 (1990). NEQUEB 10.1227/00006123-199009000-00004 2234328

[r61] Magnaes B., “Body position and cerebrospinal fluid pressure. Part 1: clinical studies on the effect of rapid postural changes,” J. Neurosurg. 44, 687–697 (1976). JONSAC 0022-3085 10.3171/jns.1976.44.6.0687 1271089

[r62] Piper I. R. et al., “An experimental study of cerebrovascular resistance, pressure transmission, and craniospinal compliance,” Neurosurgery 32, 805–816, discussion 15–16 (1993). NEQUEB 10.1227/00006123-199305000-00014 8492856

[r63] Rosner M. J. Becker D. P., “Origin and evolution of plateau waves. Experimental observations and a theoretical model,” J. Neurosurg. 60, 312–324 (1984). JONSAC 0022-3085 10.3171/jns.1984.60.2.0312 6693959

[64] Steinmeier R. et al., “Slow rhythmic oscillations of blood pressure, intracranial pressure, microcirculation, and cerebral oxygenation. Dynamic interrelation and time course in humans,” Stroke 27, 2236–2243 (1996). SJCCA7 0039-2499 10.1161/01.STR.27.12.2236 8969787

[65] Einhäupl K. M. et al., “Oscillations of ICP related to cardiovascular parameters,” in Intracranial Pressure VI, Miller J. D. Rowan J. O., Eds., pp. 290–297, Springer-Verlag, Berlin, Heidelberg, (1986).

[r66] Hashimoto M. et al., “Respiratory and cardiovascular oscillations during B-waves,” in Intracranial Pressure VII, Hoff J. T. Betz A. L., Eds., pp. 217–279, Springer, Berlin, Heidelberg (1989).

[r67] Higashi S. et al., “The role of vasomotor center and adrenergic pathway in B-waves,” in Intracranial Pressure VII, Hoff J. T. Betz A. L., Eds., pp. 220–224, Springer, Berlin, Heidelberg (1989).

[68] Venes J. L., “B waves—a reflection of cardiorespiratory or cerebral nervous systems rhythm?” Childs Brain 5, 352–360 (1979). 10.1159/000119831 456109

[69] Birn R. M. et al., “Separating respiratory-variation-related fluctuations from neuronal-activity-related fluctuations in fMRI,” Neuroimage 31, 1536–1548 (2006). NEIMEF 1053-8119 10.1016/j.neuroimage.2006.02.048 16632379

[70] Wise R. G. et al., “Resting fluctuations in arterial carbon dioxide induce significant low frequency variations in BOLD signal,” Neuroimage 21, 1652–1664 (2004). NEIMEF 1053-8119 10.1016/j.neuroimage.2003.11.025 15050588

[71] Peng T. et al., “The effects of respiratory ${\mathrm{CO}}_{2}$ fluctuations in the resting-state BOLD signal differ between eyes open and eyes closed,” Magn. Reson. Imaging 31, 336–345 (2013). MRIMDQ 0730-725X 10.1016/j.mri.2012.06.013 22921940

[72] Spiegelberg A. Preuß M. Kurtcuoglu V., “B-waves revisited,” Interdiscip. Neurosurg. 6, 13–17 (2016). 10.1016/j.inat.2016.03.004

[73] Farkas E. Luiten P. G. M., “Cerebral microvascular pathology in aging and Alzheimer’s disease,” Prog. Neurobiol. 64, 575–611 (2001). PGNBA5 0301-0082 10.1016/S0301-0082(00)00068-X 11311463

[74] Zhang R. et al., “Autonomic neural control of dynamic cerebral autoregulation in humans,” Circulation 106, 1814–1820 (2002). CIRCAZ 0009-7322 10.1161/01.CIR.0000031798.07790.FE 12356635

[75] Nafz B. Wagner C. D. Persson P. B., “Endogenous nitric oxide buffers blood pressure variability between 0.2 and 0.6 Hz in the conscious rat,” Am. J. Physiol. 272(2), H632–H637 (1997). AJPHAP 0002-9513 10.1152/ajpheart.1997.272.2.H632 9124419

[76] Rucker M. et al., “Vasomotion in critically perfused muscle protects adjacent tissues from capillary perfusion failure,” Am. J. Physiol. Heart Circ. Physiol. 279(2), H550–H558 (2000). 10.1152/ajpheart.2000.279.2.H550 10924053

[77] Chapuis B. et al., “Linear modelling analysis of baroreflex control of arterial pressure variability in rats,” J. Physiol. 559, 639–649 (2004). JPHYA7 0022-3751 10.1113/jphysiol.2004.065474 15235092 PMC1665118

[78] Diehl R. R. et al., “Phase relationship between cerebral blood flow velocity and blood pressure. A clinical test of autoregulation,” Stroke 26, 1801–1804 (1995). SJCCA7 0039-2499 10.1161/01.STR.26.10.1801 7570728

[79] Katura T. et al., “Quantitative evaluation of interrelations between spontaneous low-frequency oscillations in cerebral hemodynamics and systemic cardiovascular dynamics,” Neuroimage 31(4), 1592–1600 (2006). NEIMEF 1053-8119 10.1016/j.neuroimage.2006.02.010 16549367

[80] Obrig H. et al., “Spontaneous low frequency oscillations of cerebral hemodynamics and metabolism in human adults,” Neuroimage 12, 623–639 (2000). NEIMEF 1053-8119 10.1006/nimg.2000.0657 11112395

[81] Strangman G. Boas D. A. Sutton J. P., “Non-invasive neuroimaging using near-infrared light,” Biol. Psychiatry. 52(7), 679–693 (2002). BIPCBF 0006-3223 10.1016/S0006-3223(02)01550-0 12372658

[82] Thomsen L. L. Iversen H. K., “Experimental and biological variation of three-dimensional transcranial Doppler measurements,” J. Appl. Physiol. 75, 2805–2810 (1993). 10.1152/jappl.1993.75.6.2805 7907324

[83] Hoiland R. L. Ainslie P. N., “CrossTalk proposal: the middle cerebral artery diameter does change during alterations in arterial blood gases and blood pressure,” J. Physiol. 594, 4073–4075 (2016). JPHYA7 0022-3751 10.1113/tjp.2016.594.issue-15 27010010 PMC4806217

[84] Sarkar S. et al., “Role of transcranial Doppler ultrasonography in stroke,” Postgrad. Med. J. 83(985), 683–689 (2007). 10.1136/pgmj.2007.058602 17989267 PMC2659960

[85] Gagnon L. et al., “Further improvement in reducing superficial contamination in NIRS using double short separation measurements,” Neuroimage 85(Pt1), 127–135 (2014). NEIMEF 1053-8119 10.1016/j.neuroimage.2013.01.073 23403181 PMC3665655

[86] Obrig H., “NIRS in clinical neurology—a ‘promising’ tool?” Neuroimage 85(Pt1), 535–546 (2014). NEIMEF 1053-8119 10.1016/j.neuroimage.2013.03.045 23558099

[87] Chacon M. et al., “Comparing models of spontaneous variations, maneuvers and indexes to assess dynamic cerebral autoregulation,” Acta Neurochir. Suppl. 126, 159–162 (2018). 10.1007/978-3-319-65798-1 29492553

[88] Czosnyka M. et al., “Monitoring of cerebral autoregulation in head-injured patients,” Stroke 27, 1829–1834 (1996). SJCCA7 0039-2499 10.1161/01.STR.27.10.1829 8841340

[89] Steiner L. A. et al., “Near-infrared spectroscopy can monitor dynamic cerebral autoregulation in adults,” Neurocrit. Care 10, 122–128 (2009). 10.1007/s12028-008-9140-5 18807218

[90] Liu X. et al., “Comparison of frequency and time domain methods of assessment of cerebral autoregulation in traumatic brain injury,” J. Cereb. Blood Flow Metab. 35, 248–256 (2015). 10.1038/jcbfm.2014.192 25407266 PMC4426741

[91] Zhang R. et al., “Transfer function analysis of dynamic cerebral autoregulation in humans,” Am. J. Physiol. 274, H233–H241 (1998). 10.1152/ajpheart.1998.274.1.H233 9458872

[92] Shiogai Y. Stefanovska A. McClintock P. V., “Nonlinear dynamics of cardiovascular ageing,” Phys. Rep. 488, 51–110 (2010). 10.1016/j.physrep.2009.12.003 20396667 PMC2853263

[93] Bruns A., “Fourier-, Hilbert- and wavelet-based signal analysis: are they really different approaches?” J. Neurosci. Methods 137, 321–332 (2004). 10.1016/j.jneumeth.2004.03.002 15262077

[94] Thewissen L. et al., “Measuring near-infrared spectroscopy derived cerebral autoregulation in neonates: from research tool toward bedside multimodal monitoring,” Front. Pediatr. 6, 117 (2018). 10.3389/fped.2018.00117 29868521 PMC5960703

[95] Strangman G. Franceschini M. A. Boas D. A., “Factors affecting the accuracy of near-infrared spectroscopy concentration calculations for focal changes in oxygenation parameters,” NeuroImage 18, 865–879 (2003). NEIMEF 1053-8119 10.1016/S1053-8119(03)00021-1 12725763

[96] Smielewski P. et al., “Can cerebrovascular reactivity be measured with near-infrared spectroscopy?,” Stroke 26(12), 2285–2292 (1995). SJCCA7 0039-2499 10.1161/01.STR.26.12.2285 7491652

[97] Strangman G. et al., “A quantitative comparison of simultaneous BOLD fMRI and NIRS recordings during functional brain activation,” Neuroimage 17, 719–731 (2002). NEIMEF 1053-8119 10.1006/nimg.2002.1227 12377147

[98] Reinhard M. et al., “Oscillatory cerebral hemodynamics—the macro- vs. microvascular level,” J. Neurol. Sci. 250, 103–109 (2006). 10.1016/j.jns.2006.07.011 17011584

[99] Phillip D. et al., “Low frequency oscillations in cephalic vessels assessed by near infrared spectroscopy,” Eur. J. Clin. Invest. 42, 1180–1188 (2012). CECED9 10.1111/eci.2012.42.issue-11 22897146 PMC3730271

[100] Cheng R. et al., “Noninvasive optical evaluation of spontaneous low frequency oscillations in cerebral hemodynamics,” Neuroimage 62, 1445–1454 (2012). NEIMEF 1053-8119 10.1016/j.neuroimage.2012.05.069 22659481

[101] Pfurtscheller G. et al., “About the stability of phase shifts between slow oscillations Around 0.1 Hz in cardiovascular and cerebral systems,” IEEE Trans. Biomed. Eng. 58, 2064–2071 (2011). 10.1109/TBME.2011.2134851 21693389

[102] Bu L. et al., “Effects of poor sleep quality on brain functional connectivity revealed by wavelet-based coherence analysis using NIRS methods in elderly subjects,” Neurosci. Lett. 668, 108–114 (2018). 10.1016/j.neulet.2018.01.026 29353214

[103] Bu L. et al., “Effects of sleep deprivation on phase synchronization as assessed by wavelet phase coherence analysis of prefrontal tissue oxyhemoglobin signals,” PLoS One 12, e0169279 (2017). POLNCL 1932-6203 10.1371/journal.pone.0169279 28046043 PMC5207699

[104] Li Z. et al., “Wavelet analysis of cerebral oxygenation signal measured by near infrared spectroscopy in subjects with cerebral infarction,” Microvasc. Res. 80, 142–147 (2010). MIVRA6 0026-2862 10.1016/j.mvr.2010.02.004 20156461

[105] Phillip D. et al., “Spontaneous low frequency oscillations in acute ischemic stroke: a near infrared spectroscopy (NIRS) study,” J. Neurol. Neurophysiol. 5, 1000241 (2014). 10.4172/2155-9562

[106] Dorrance A. M. Fink G., “Effects of stroke on the autonomic nervous system,” Compr. Physiol. 5, 1241–1263 (2015). 10.1002/cphy.c140016 26140717

[107] Schroeter M. L. et al., “Spontaneous slow hemodynamic oscillations are impaired in cerebral microangiopathy,” J. Cereb. Blood Flow Metab. 25, 1675–1684 (2005). 10.1038/sj.jcbfm.9600159 15931161

[108] van Beek A. H. et al., “Oscillations in cerebral blood flow and cortical oxygenation in Alzheimer’s disease,” Neurobiol. Aging 33, 428.e21–428.e31 (2012). 10.1016/j.neurobiolaging.2010.11.016 21208686

[109] Aharon-Peretz J. et al., “Increased sympathetic and decreased parasympathetic cardiac innervation in patients with Alzheimer’s disease,” Arch. Neurol. 49, 919–922 (1992). ARNEAS 0003-9942 10.1001/archneur.1992.00530330041013 1520081

[r110] de Vilhena Toledo M. A. Junqueira L. F. Jr., “Cardiac sympathovagal modulation evaluated by short-term heart interval variability is subtly impaired in Alzheimer’s disease,” Geriatr. Gerontol. Int. 8, 109–118 (2008). 10.1111/j.1447-0594.2008.00456.x 18713163

[111] Zakrzewska-Pniewska B. et al., “Clinical and functional assessment of dysautonomia and its correlation in Alzheimer’s disease,” Am. J. Alzheimers Dis. Other Demen. 27, 592–599 (2012). 10.1177/1533317512459792 23007287 PMC10845696

[112] Han Q. et al., “Phase synchronization analysis of prefrontal tissue oxyhemoglobin oscillations in elderly subjects with cerebral infarction,” Med. Phys. 41, 102702 (2014). 10.1118/1.4896113 25281981

[r113] Han Q. et al., “Wavelet coherence analysis of prefrontal tissue oxyhaemoglobin signals as measured using near-infrared spectroscopy in elderly subjects with cerebral infarction,” Microvasc. Res. 95, 108–115 (2014). MIVRA6 0026-2862 10.1016/j.mvr.2014.08.001 25117487

[114] Tan Q. et al., “Frequency-specific functional connectivity revealed by wavelet-based coherence analysis in elderly subjects with cerebral infarction using NIRS method,” Med. Phys. 42, 5391–5403 (2015). 10.1118/1.4928672 26328988

[115] Phillip D. et al., “Altered low frequency oscillations of cortical vessels in patients with cerebrovascular occlusive disease—a NIRS study,” Front. Neurol. 4, 204 (2013). 10.3389/fneur.2013.00204 24379801 PMC3864103

[116] Kuroda S. Houkin K., “Moyamoya disease: current concepts and future perspectives,” Lancet Neurol. 7, 1056–1066 (2008). 10.1016/S1474-4422(08)70240-0 18940695

[117] He Y. et al., “Wavelet analysis of cerebral oxygenation oscillations in the screening of Moyamoya disease,” Biomed. Mater. Eng. 24, 3463–3469 (2014). 10.3233/BME-141171 25227058

[118] Fukui M. et al., “Moyamoya disease,” Neuropathology 20(Suppl 1), 61–64 (2000). NANEDL 1365-2990 10.1046/j.1440-1789.2000.00300.x 11037190

[119] Schytz H. W. et al., “Nitric oxide modulation of low-frequency oscillations in cortical vessels in FHM—a NIRS study,” Headache 52, 1146–1154 (2012). HEADAE 0017-8748 10.1111/hed.2012.52.issue-7 22352839 PMC3730264

[120] Schytz H. W. et al., “Low-frequency oscillations and vasoreactivity of cortical vessels in obstructive sleep apnea during wakefulness: a near infrared spectroscopy study,” Sleep Med. 14, 416–421 (2013). 10.1016/j.sleep.2012.12.009 23517585 PMC6420812

[121] Kohler M. Stradling J. R., “Mechanisms of vascular damage in obstructive sleep apnea,” Nat. Rev. Cardiol. 7(12), 677–685 (2010). 10.1038/nrcardio.2010.145 21079639

[122] Kolyva C. et al., “Oscillations in cerebral haemodynamics in patients with falciparum malaria,” Adv. Exp. Med. Biol. 765, 101–107 (2013). AEMBAP 0065-2598 10.1007/978-1-4614-4989-8 22879021 PMC4038006

[123] Mishra S. K. Newton C. R., “Diagnosis and management of the neurological complications of falciparum malaria,” Nat. Rev. Neurol. 5, 189–198 (2009). 10.1038/nrneurol.2009.23 19347024 PMC2859240

[124] Reinhard M. et al., “Spatial mapping of dynamic cerebral autoregulation by multichannel near-infrared spectroscopy in high-grade carotid artery disease,” J. Biomed. Opt. 19, 097005 (2014). 10.1117/1.JBO.19.9.097005 25253194

[125] Muehlschlegel S. et al., “Feasibility of NIRS in the neurointensive care unit: a pilot study in stroke using physiological oscillations,” Neurocrit. Care 11, 288–295 (2009). 10.1007/s12028-009-9254-4 19649749 PMC2782535

[126] Oldag A. et al., “Near-infrared spectroscopy and transcranial sonography to evaluate cerebral autoregulation in middle cerebral artery steno-occlusive disease,” J. Neurol. 263, 2296–2301 (2016). 10.1007/s00415-016-8262-5 27544503

[127] Bos M. J. et al. “Transcranial Doppler hemodynamic parameters and risk of stroke: the Rotterdam study,” Stroke 38, 2453–2458 (2007). SJCCA7 0039-2499 10.1161/STROKEAHA.107.483073 17673712

[128] Li Z. et al., “Spectral analysis of near-infrared spectroscopy signals measured from prefrontal lobe in subjects at risk for stroke,” Med. Phys. 39, 2179–2185 (2012). MPHYA6 0094-2405 10.1118/1.3696363 22482639

[129] Li Z. et al., “Assessment of cerebral oxygenation oscillations in subjects with hypertension,” Microvasc. Res. 88, 32–41 (2013). MIVRA6 0026-2862 10.1016/j.mvr.2013.04.003 23583904

[130] Zeller J. B. M. et al., “Reduced spontaneous low frequency oscillations as measured with functional near-infrared spectroscopy in mild cognitive impairment,” Brain Imaging Behav. 1–10 (2018). 10.1007/s11682-018-9827-y 29362991

[131] Schroeter M. L. Schmiedel O. von Cramon D. Y., “Spontaneous low-frequency oscillations decline in the aging brain,” J. Cereb. Blood Flow Metab. 24, 1183–1191 (2004). 10.1097/01.WCB.0000135231.90164.40 15529019

[132] Li Z. et al., “Age-related changes in spontaneous oscillations assessed by wavelet transform of cerebral oxygenation and arterial blood pressure signals,” J. Cereb. Blood Flow Metab. 33, 692–699 (2013). 10.1038/jcbfm.2013.4 23361392 PMC3652694

[133] Song S. et al., “Low-frequency oscillations in cerebrovascular and cardiovascular hemodynamics: their interrelationships and the effect of age, Microvasc. Res. 102, 46–53 (2015). MIVRA6 0026-2862 10.1016/j.mvr.2015.08.004 26277229

[134] Tan Q. et al., “Age-related alterations in phase synchronization of oxyhemoglobin concentration changes in prefrontal tissues as measured by near-infrared spectroscopy signals,” Microvasc. Res. 103, 19–25 (2016). MIVRA6 0026-2862 10.1016/j.mvr.2015.10.002 26525098

[135] Vermeij A. et al., “Very-low-frequency oscillations of cerebral hemodynamics and blood pressure are affected by aging and cognitive load,” Neuroimage 85(Pt1), 608–615 (2014). 10.1016/j.neuroimage.2013.04.107 23660026

[136] Peng T. et al., “The effects of age on the spontaneous low-frequency oscillations in cerebral and systemic cardiovascular dynamics,” Physiol. Meas. 29, 1055–1069 (2008). PMEAE3 0967-3334 10.1088/0967-3334/29/9/005 18756026

[137] Oudegeest-Sander M. H. et al., “Assessment of dynamic cerebral autoregulation and cerebrovascular ${\mathrm{CO}}_{2}$ reactivity in ageing by measurements of cerebral blood flow and cortical oxygenation,” Exp. Physiol. 99, 586–598 (2014). EXPHEZ 0958-0670 10.1113/expphysiol.2013.076455 24363382

[138] Li Z. et al., “Wavelet coherence analysis of prefrontal oxygenation signals in elderly subjects with hypertension,” Physiol. Meas. 35, 777–791 (2014). PMEAE3 0967-3334 10.1088/0967-3334/35/5/777 24670282

[139] Gao Y. et al., “Cerebral autoregulation in response to posture change in elderly subjects-assessment by wavelet phase coherence analysis of cerebral tissue oxyhemoglobin concentrations and arterial blood pressure signals,” Behav. Brain Res. 278, 330–336 (2015). BBREDI 0166-4328 10.1016/j.bbr.2014.10.019 25453742

[140] Cui R. et al., “Wavelet coherence analysis of spontaneous oscillations in cerebral tissue oxyhemoglobin concentrations and arterial blood pressure in elderly subjects,” Microvasc. Res. 93, 14–20 (2014). MIVRA6 0026-2862 10.1016/j.mvr.2014.02.008 24594440

[141] Wang B. et al., “Posture-related changes in brain functional connectivity as assessed by wavelet phase coherence of NIRS signals in elderly subjects,” Behav. Brain Res. 312, 238–245 (2016). BBREDI 0166-4328 10.1016/j.bbr.2016.06.037 27335218

[142] Kainerstorfer J. M. Sassaroli A. Fantini S., “Optical oximetry of volume-oscillating vascular compartments: contributions from oscillatory blood flow,” J. Biomed. Opt. 21, 101408 (2016). JBOPFO 1083-3668 10.1117/1.JBO.21.10.101408 26926870 PMC4772448

[143] Scholkmann F. et al., “A review on continuous wave functional near-infrared spectroscopy and imaging instrumentation and methodology,” Neuroimage 85(Pt1), 6–27 (2014). NEIMEF 1053-8119 10.1016/j.neuroimage.2013.05.004 23684868

[144] Tzeng Y. C. Panerai R. B., “CrossTalk proposal: dynamic cerebral autoregulation should be quantified using spontaneous blood pressure fluctuations,” J. Physiol. 596, 3–5 (2018). JPHYA7 0022-3751 10.1113/JP273899 29207213 PMC5746519

